# Supplementary material for: HIVprotI: an integrated web based platform for prediction and design of HIV proteins inhibitors
Source: J Cheminform. 2018 Mar 9;10:12. doi: 10.1186/s13321-018-0266-y (PMC5845081; doi:10.1186/s13321-018-0266-y)
Supplement: Supplementary file 2 — Additional file 2. Source code of HIVProtI web server. [file 13321_2018_266_MOESM2_ESM.zip › HIVprotI_Source-code/hivprotI/weka-3-6-6/doc/weka/classifiers/rules/part/C45PruneableDecList.html]

C45PruneableDecList


---


|  |  |  |
| --- | --- | --- |
| **Overview** \| **Package** \| **Class** \| **Tree** \| **Deprecated** \| **Index** \| **Help** \| **Weka's home** | |  |
| PREV CLASS   **NEXT CLASS** | **FRAMES**    **NO FRAMES**     **All Classes** |  |
| SUMMARY: NESTED \| FIELD \| CONSTR \| METHOD | DETAIL: FIELD \| CONSTR \| METHOD |  |


---


## weka.classifiers.rules.part Class C45PruneableDecList

```
java.lang.Object
  weka.classifiers.rules.part.ClassifierDecList
      weka.classifiers.rules.part.C45PruneableDecList
```

**All Implemented Interfaces:**: java.io.Serializable, RevisionHandler

---

``` public class C45PruneableDecList extends ClassifierDecList ```

Class for handling a partial tree structure pruned using C4.5's
pruning heuristic.

**Version:**
:   $Revision: 1.9 $

**Author:**
:   Eibe Frank (eibe@cs.waikato.ac.nz)

**See Also:**: Serialized Form

---

**Constructor Summary**

`C45PruneableDecList(ModelSelection toSelectLocModel,
double cf,
int minNum)`
  
          Constructor for pruneable tree structure.


| **Method Summary** | |
| --- | --- |
| `void` | `buildDecList(Instances data, boolean leaf)`             Builds the partial tree without hold out set. |
| `double` | `getEstimatedErrorsForLeaf()`             Computes estimated errors for leaf. |
| `java.lang.String` | `getRevision()`             Returns the revision string. |

**Methods inherited from class weka.classifiers.rules.part.ClassifierDecList**

`buildRule, chooseIndex, chooseLastIndex, classifyInstance, cleanup, distributionForInstance, toString, weight`

**Methods inherited from class java.lang.Object**

`equals, getClass, hashCode, notify, notifyAll, wait, wait, wait`

**Constructor Detail**

### C45PruneableDecList

```
public C45PruneableDecList(ModelSelection toSelectLocModel,
                           double cf,
                           int minNum)
                    throws java.lang.Exception
```

:   Constructor for pruneable tree structure. Stores reference
    to associated training data at each node.

    **Parameters:**: `toSelectLocModel` - selection method for local splitting model: `cf` - the confidence factor for pruning: `minNum` - the minimum number of objects in a leaf **Throws:**: `java.lang.Exception` - if something goes wrong


**Method Detail**

### buildDecList

```
public void buildDecList(Instances data,
                         boolean leaf)
                  throws java.lang.Exception
```

:   Builds the partial tree without hold out set.

    :   **Overrides:**: `buildDecList` in class `ClassifierDecList`
    :   **Throws:**: `java.lang.Exception` - if something goes wrong

---


### getEstimatedErrorsForLeaf

```
public double getEstimatedErrorsForLeaf()
```

:   Computes estimated errors for leaf.

---


### getRevision

```
public java.lang.String getRevision()
```

:   Returns the revision string.

    :   **Specified by:**: `getRevision` in interface `RevisionHandler` **Overrides:**: `getRevision` in class `ClassifierDecList`
    :   **Returns:**: the revision


---


|  |  |  |
| --- | --- | --- |
| **Overview** \| **Package** \| **Class** \| **Tree** \| **Deprecated** \| **Index** \| **Help** \| **Weka's home** | |  |
| PREV CLASS   **NEXT CLASS** | **FRAMES**    **NO FRAMES**     **All Classes** |  |
| SUMMARY: NESTED \| FIELD \| CONSTR \| METHOD | DETAIL: FIELD \| CONSTR \| METHOD |  |


---
